# Intermittent Fever in Chelsea.— General Summary of the Facts Contained in the Report, and the Inferences Therefrom

**Published:** 1853-03

**Authors:** 


*Intermittent Fever in Chelsea.—General Summary of the Facts contained in the Report, and the Inferences therefrom.*

*First.*—In 1789 a dam was erected at Chelsea, whereby the salt water was cut off from two hundred and seventy-five acres of land adjacent to the village. From this period, until 1816, various ineffectual endeavors were made to reduce this tract to cultivation. Finally, this plan was given up in despair, and the water again allowed to overflow the land; but of course it could not do so as easily and effectually as before, owing to the fact that there was only a narrow sluice-gate through which the tide was compelled to ebb and flow. In 1845 the gate was again closed, and has remained so ever since, whereby the old bed of the river and a large space of porous earth were left exposed to the rays of the sun and with a southern exposure.

*Second.*—It has been proved that certainly three, and probably four, cases of genuine fever and ague have arisen either on, or very near, the edge of the partially dried up marsh, and this since 1845, when the dam was finally closed. As these are the only well-authenticated cases* of indigenous fever and ague in this vicinity, and as similar results from similar causes have occurred elsewhere in Massachusetts, we infer that the dam has been the exciting cause of their appearance.

*Third.*—Chelsea has been more liable to dysentery than other places, as far back as any statistics will carry us, and since 1845, this tendency has been augmented. From these statistics we learn that Chelsea is about three times as subject to the malady in question as Boston, and twice as much as the state at large, Lynn and Gloucester.

*Fourth.*—Notwithstanding Chelsea is thus subject to the influence of fever and ague and dysentery, it is not a more unhealthy place of residence than the other towns with which it has been compared. The statistics of table 2 prove this.

*Fifth.*—May not the curious fact that Chelsea is more liable to fatal dysentery than other towns, while, at the same time, its annual percentage of deaths to the population is only equal to, and in some respects less than, the annual percentages of these same towns; may not this fact be an illustration of the great law of compensation which governs the liability of communities and nations to disease and death? Does it not point to the inference, that while certain influences tend to excite certain diseases, these same influences may check the development of others?

*Sixth.*—One final inference, of a very evident and practical character, the committee would make in concluding their report. It is as follows: Whenever any patient in Chelsea seems to have a tendency to diseases of an intermittent type or to dysentery, it becomes the duty of the medical attendant

---

* See exceptions to this. [See Appendix.]

to think of the propriety of removing him from the deleterious influences exerted on him.

HENRY I. BOWDITCH,
JOHN WARE,
*February*, 1852. EPHRAIM BUCK.

*Appendix.*—After this report was presented to the Society, the committee were informed of the following very important case. By the kindness of Dr. W. H. Thayer, the committee are enabled to present it, as drawn up by him from his original notes:

*Sept.* 30, 1850.—John Mason, æt. 12. School-boy. Eliot-st. Has lived with his present guardian since six months old. Birthplace unknown. Has not been out of city over night since 2 years old. Always lived where now does, between Washington and Tremont streets.

Rather puny. Witnessed display of fireworks on 18th inst., on the common, sitting on the ground. The evening was too cool to sit in the house with open window, and there was a dense fog. The next day, having been previously well, had a chill in the afternoon, followed by heat, and that again by sweating. The next day (20th) he visited the Mechanics' Exhibition, was much fatigued, and had a more severe exacerbation of the same character than the day before.

The same attack has been repeated every day since, the chill being from 5 to 6½, P. M., followed by great heat, which ends by profuse sweating at 11, P. M. With the paroxysm comes headache, pain in neck and across small of back. At the first attack he had nausea—none since. He has lost strength and appetite, is very thirsty during heat; is up all day.

Now—Sept. 30th, 1, P. M. Up and dressed. Pale; cool; p. 70; tongue nearly clean; has no pain. During paroxysm, has some cough, which gives him a little pain in chest. None now. Nothing abnormal discovered on auscultation. Has taken a cathartic since first attack. No other treatment. One dejection daily.

℞. Quiniæ sulphatis, gr. jss.;
Acidi tartarici, gr. ⅗. M.

Three times daily, avoiding paroxysm. Cold sponging during heat.

Paroxysms occurred as usual on the 30th of Sept. and 1st of October. On the first, without any pain in neck and loins.

On the 2d October, chill from 9¼ to 10, P. M., followed by heat till 2, A.M. Pain in neck and back severe. On the 3d October, no chill—but heat from 1 to 5, A. M., of October 4th. Quiniæ s., gr. ij. four times daily.

*Oct. 5th.*—No chill nor heat. Sweating began at 2, A. M., and was profuse.

*6th.*—No chill, heat nor sweat. Feels well. Spleen cannot be felt; no tenderness over it. There was some tenderness across upper abdomen at the first visit, but spleen was not searched for. One dej. daily. Pill three times a day.

*9th.*—No chill nor heat since last visit. Sweats freely every night, but this is his habit. Is at school. Mother reports him very well.

Omit medicine.

By the following still more curious document, brought to the notice of the committee by Dr. Z. B. Adams, we have undoubted proof that genuine intermittent fever formerly prevailed, and was quite fatal on some parts of the eastern shores of Massachusetts.

These data are extracted from a diary kept by the Rev. Noadiah Russell, in 1682 and 3, while a tutor at Harvard College. By the College Catalogue this gentleman appears to have received his degree of A. B. in 1681. He died in 1713. When writing his diary of everyday events, he scarcely could have hoped to have been the means of illustrating the history of disease in New England. The original diary, fragments of which alone remain, is now in the possession of his descendants, and has been recently published. (See Drake's New England Historical and Genealogical Register, &c., January, 1853, page 53.)

"16th day 6 month (Aug. 16, 1682). * * The next day being Fryday I went to wait on some company to Lynspring where for company's sake drinking too much cold water I set myself in an ague wch came again on Sabbath day and Tuesday.

"10 6 (Aug. 10, 1683) Samuel Gardner a student of ye College, of 2 years standing prompt for learning exemplary for piety and Sobriety died at Salem of ye feaver at which time many were visited with ye feaver and ague which was very mortal."—*Boston Med. and Surg. Journal.*

